# Supplementary figures and images for: Cell-free tumour DNA analysis detects copy number alterations in gastro-oesophageal cancer patients
Source: PLoS One. 2021 Feb 4;16(2):e0245488. doi: 10.1371/journal.pone.0245488 (PMC7861431; doi:10.1371/journal.pone.0245488)

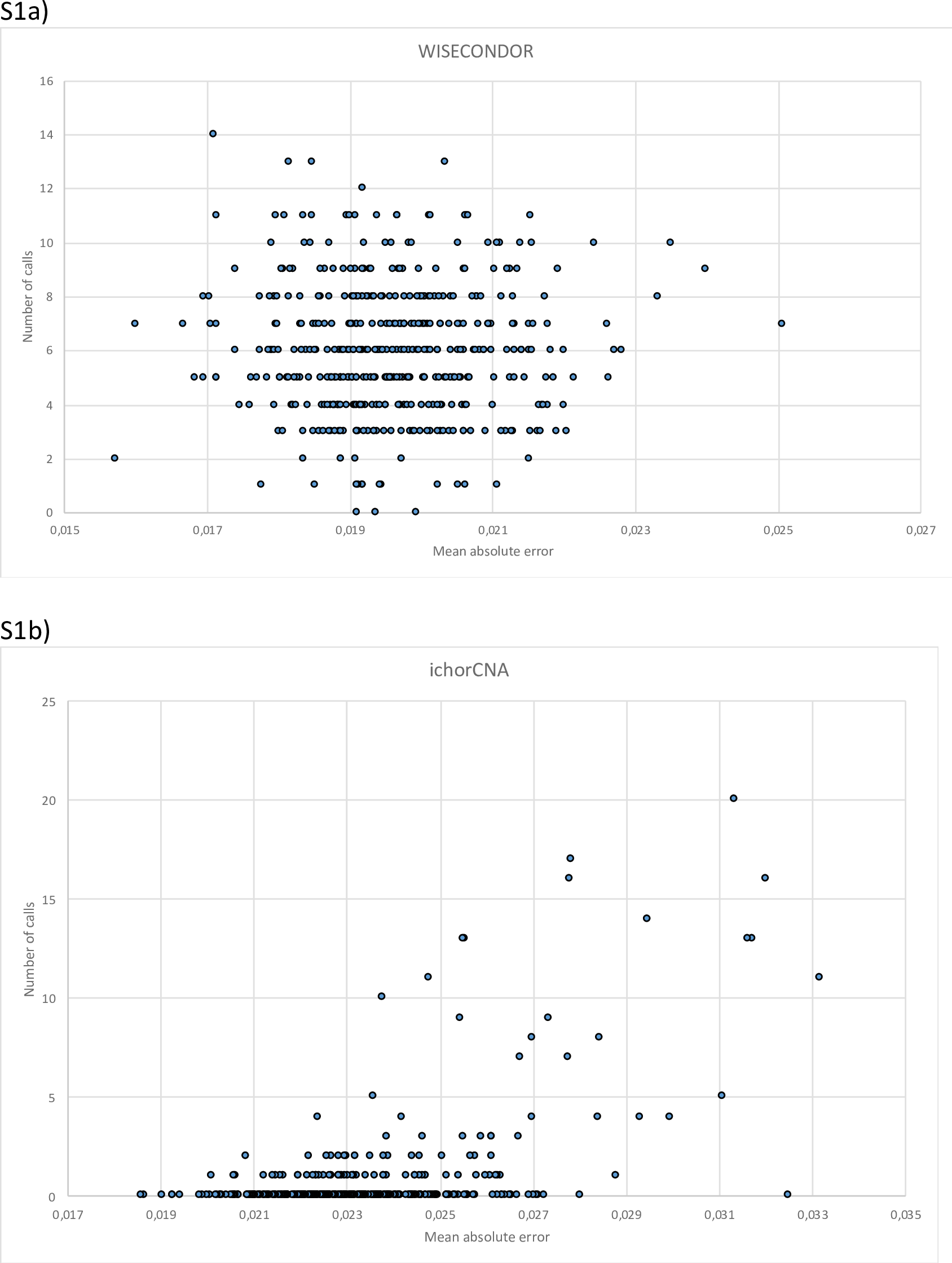

Supplement: S1 Fig — Mean absolute error of the normalized coverage difference between all bins and the number of calls for all of the samples in the reference set (n = 414). The mean absolute error is plotted on the X cropped axis and the number of copy number alterations called by WISECONDOR (S1a) and ichorCNA (S1b) are plotted on the Y axis. (TIF) [file pone.0245488.s001.tif]

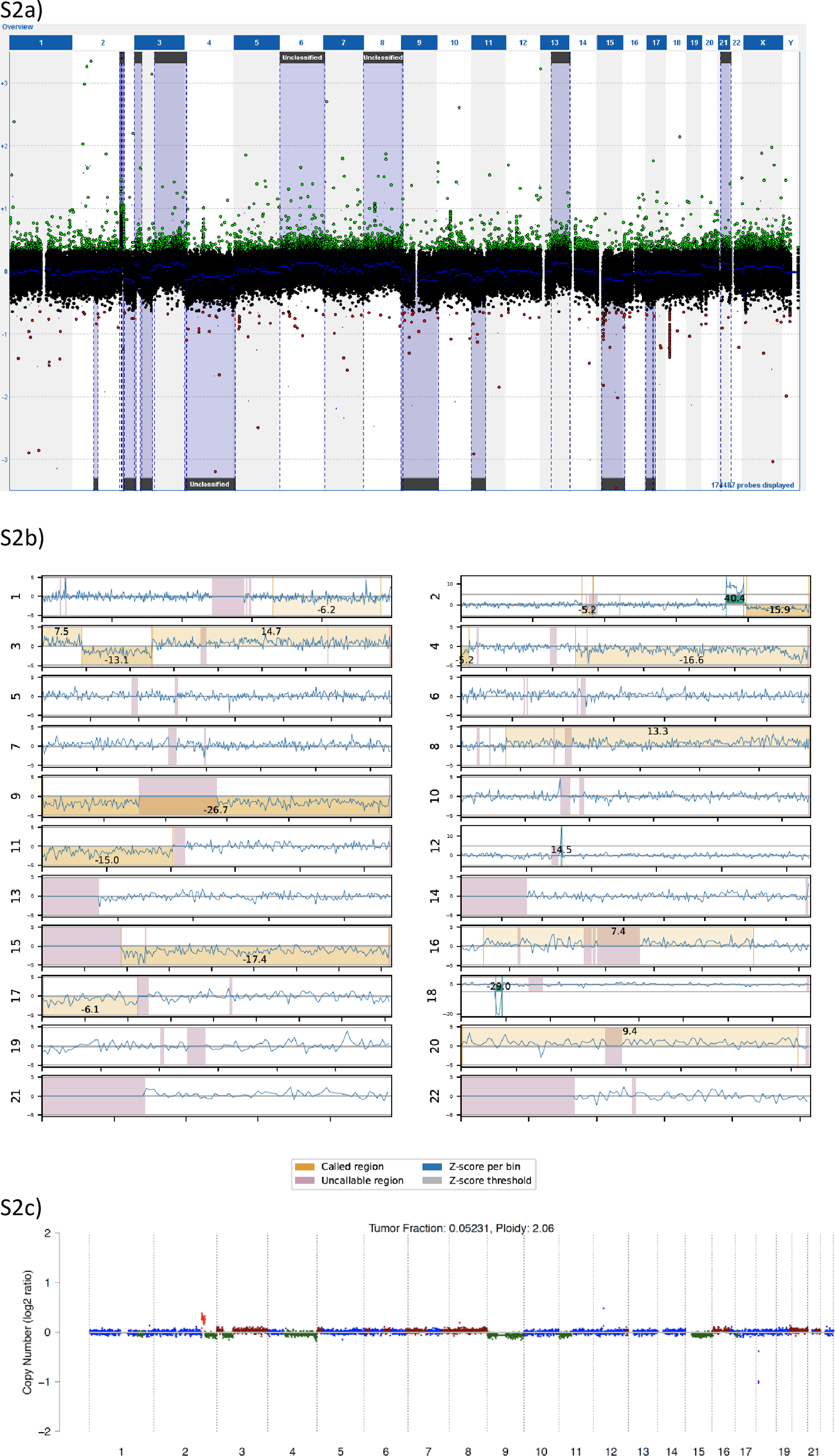

Supplement: S2 Fig — In the tissue array-CGH (S2a) copy number alterations (CNAs) after filtering are indicated by blue boxes with gains above the zero line and losses below. In the upper panel are the general overview chromosomal positions and, on the Y-axis, the log2 ratio of each probe is shown as dots. The moving average is indicated by a blue line. In the low-coverage whole-genome analysis in plasma using WISECONDOR (S2b), the blue line indicates the bin Z-score. Called regions (before filtering) are indicated by yellow/green boxes depending on the effect size together with the Z-score for the region. In the low-coverage whole-genome analysis in plasma using ichorCNA (S2c) dots represent bins with their log2 ratio shown on the Y-axis. Regions with gains, including amplifications, are indicated by brown/red colour and losses are indicated by green. (TIF) [file pone.0245488.s002.tif]

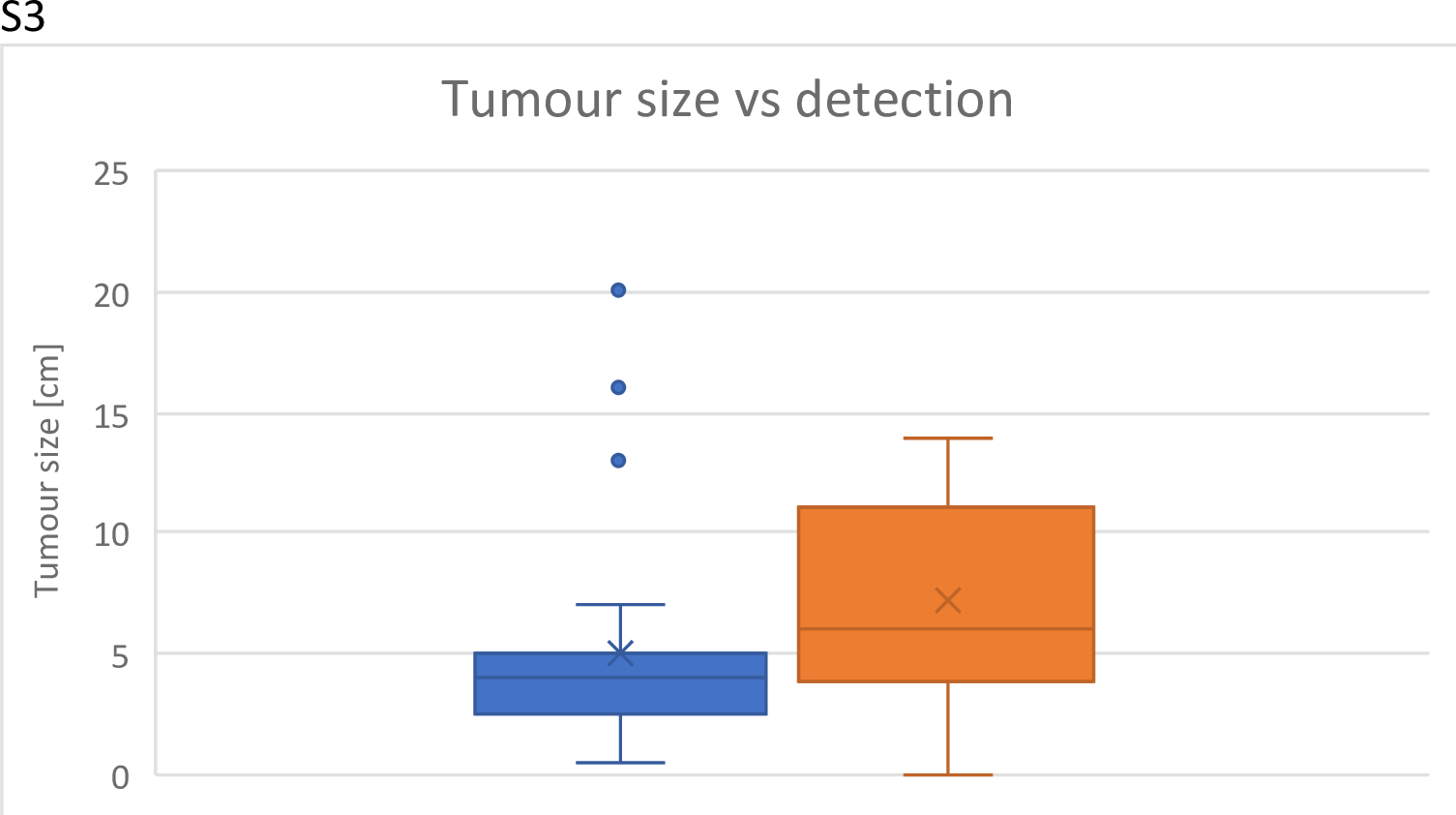

Supplement: S3 Fig — Tumour size [cm] in the group with detectable CNAs in plasma (blue box) and in the group with no detectable CNAs in plasma (orange box). Mann-Whitney, plotted in R software. p = 0.06056. (TIF) [file pone.0245488.s003.tif]

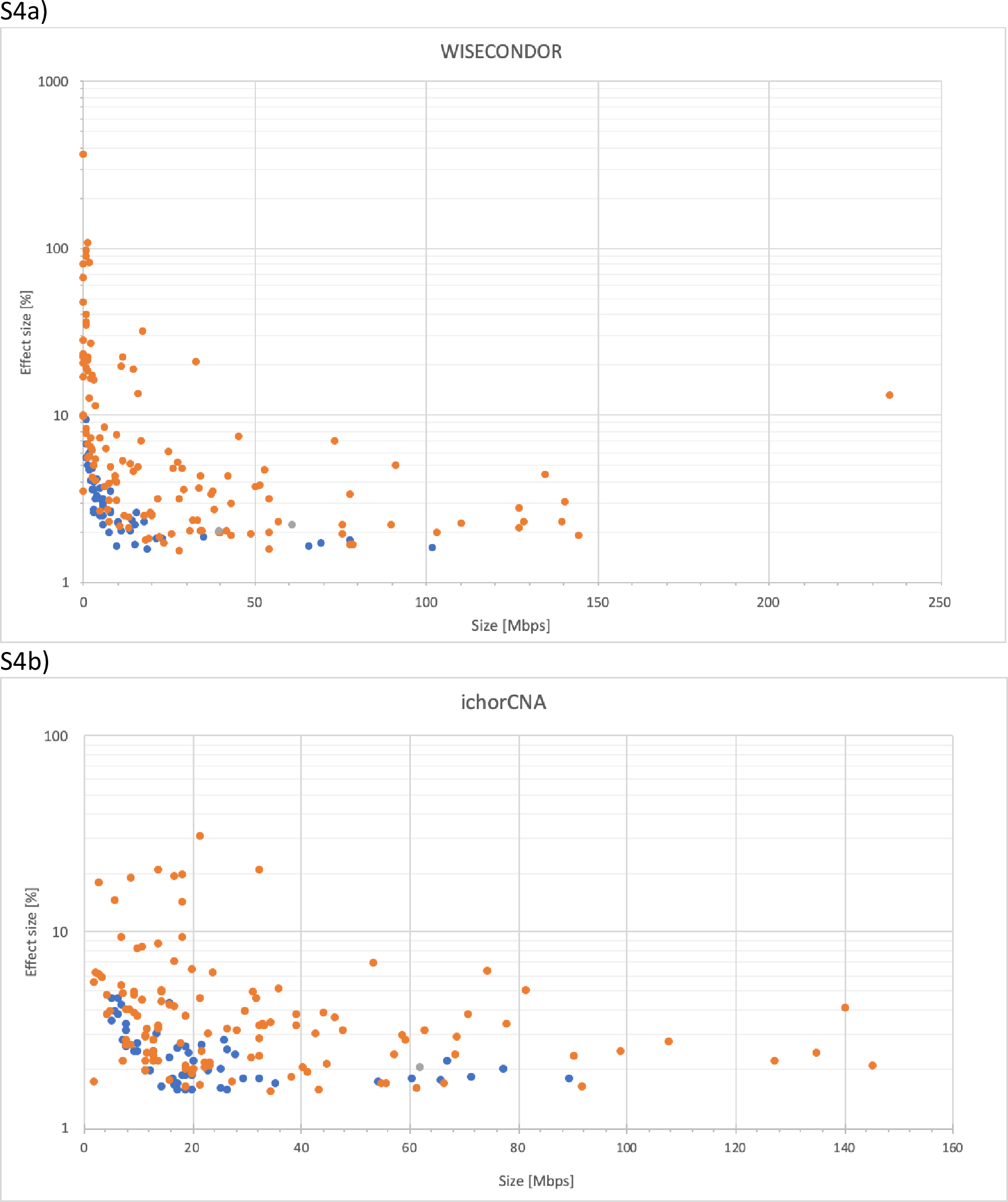

Supplement: S4 Fig — Plot of all cancer-associated CNAs in plasma from individuals (n = 13) with tissue samples taken before any chemotherapy and CNAs called by WISECONDOR (S4a) and ichor (S4b). On the log10 Y-axis, effect size of the CNA and on the X-axis, size in megabasepairs (Mbps) of the CNA. CNAs verified in the tissue array-CGH are coloured orange and the “unverified” are coloured blue. The CNAs coloured grey (n = 3 in total) were manually classified as cancer-associated even though they were not visible in the tissue array-CGH (see Methods for details). (TIF) [file pone.0245488.s004.tif]
