# Supplementary material for: Cell-free tumour DNA analysis detects copy number alterations in gastro-oesophageal cancer patients
Source: PLoS One. 2021 Feb 4;16(2):e0245488. doi: 10.1371/journal.pone.0245488 (PMC7861431; doi:10.1371/journal.pone.0245488)
Supplement: S1 Table — All 61 regions included in the targeted analysis. Cytoband and refseq gene positions are provided in genome build GRCh37/hg19. (DOCX) [file pone.0245488.s005.docx]

**S1 Table**

**Regions and genes in the targeted plasma analysis**

All 61 regions included in the targeted analysis. Cytoband and refseq gene positions are provided in genome build GRCh37/hg19.
