# Supplementary material for: Cell-free tumour DNA analysis detects copy number alterations in gastro-oesophageal cancer patients
Source: PLoS One. 2021 Feb 4;16(2):e0245488. doi: 10.1371/journal.pone.0245488 (PMC7861431; doi:10.1371/journal.pone.0245488)
Supplement: S3 Table — NIPT analysis results of plasma from 15 pregnant women carrying foetuses with trisomy 21, trisomy 18 or trisomy 13 (verified by invasive test). All trisomies were correctly detected by WISECONDOR and ichorCNA. For each sample the foetal trisomy chromosome and the estimated foetal fraction are provided in separate columns. (DOCX) [file pone.0245488.s007.docx]

**S3 Table**

**Foetal positive control NIPT samples**

NIPT analysis results of plasma from 15 pregnant women carrying foetuses with trisomy 21, trisomy 18 or trisomy 13 (verified by invasive test). All trisomies were correctly detected by WISECONDOR and ichorCNA. For each sample the foetal trisomy chromosome and the estimated foetal fraction are provided in separate columns.
